# MiR-924 as a tumor suppressor inhibits non-small cell lung cancer by inhibiting RHBDD1/Wnt/β-catenin signaling pathway

**DOI:** 10.1186/s12935-020-01516-0

**Published:** 2020-10-08

**Authors:** Huaishi Wang, Xi Chen, Baishuang Yang, Zhi Xia, Qiong Chen

**Affiliations:** 1 Department of Geriatrics, Xiangya Hospital of Central South University, NO 87 Xiangya Road, Changsha, China; 2 Respiratory Medicine, Xiangya Hospital of Central South University, Changsha, China; 3 National Clinical Research Center for Geriatric Disorders, Xiangya Hospital, Central South University, Changsha, China

**Keywords:** miR-924, RHBDD1, Non-small cell lung cancer, Wnt/β-catenin signaling

## Abstract

**Background:**

MiR-924 has been reported to be a tumor suppressor in hepatocellular carcinoma. However, the functions and mechanisms of miR-924 in non-small cell lung cancer (NSCLC) remain unclear.

**Methods:**

The expression of miR-924 was determined in NSCLC tissues and cell lines using quantitative real time PCR. The Chi-squared test was used to evaluate the correlation between miR-924 levels and clinicopathological parameters in patients with NSCLC. Cell proliferation was assessed by CCK-8 assay. Cell migration and invasion were detected by transwell assay. The combination of miR-924 and RHBDD1 was analyzed via the luciferase reporter assay. The expression level of RHBDD1 was evaluated in lung cancer tissues using public microarray datasets form Oncomine and its prognostic value was assessed by Kaplan–Meier Plotter databases. A tumor xenograft mouse model was established to illustrate the effects of miR-924 on the tumorigenesis of NSCLC in vivo.

**Results:**

In this study, we found miR-924 was strikingly decreased in NSCLC tissues and cell lines. Decreased miR-924 was closely correlated with advanced tumor-node-metastasis (TNM) stage and lymphatic metastasis in NSCLC patients. Noticeably, rhomboid domain-containing protein 1 (RHBDD1) was predicted and confirmed as a direct target of miR-924. Moreover, the expression level of RHBDD1 was significantly increased and inversely associated with prognosis using public microarray datasets form Oncomine and Kaplan–Meier Plotter databases. MiR-924 overexpression suppressed cell proliferation, migration and invasion. The in vivo experiments further demonstrated that miR-924 overexpression reduced NSCLC xenograft growth through inhibiting RHBDD1/Wnt/β-catenin signaling pathway.

**Conclusions:**

In summary, these findings demonstrated that miR-924 blocked the progression of NSCLC by targeting RHBDD1 and miR-924/RHBDD1 axis might provide a novel therapeutic target for the treatment of NSCLC.

## Background

Lung cancer is one of the most common malignancies with higher morbidity and mortality worldwide [1]. As the predominant type of lung cancer, non-small-cell lung cancer (NSCLC) includes squamous cell carcinoma, adenocarcinoma and large cell carcinoma, accounting for approximately 85% of all lung cancer cases [2]. Even though therapeutic strategies have made significant improvements over the past two decades, the 5-year survival rate of NSCLC patients still just around 15% [3]. Therefore, elucidating the underlying mechanism is urgently required for exploration of innovative therapies for NSCLC patients.

MicroRNAs (miRNAs, miRs) are a class of small noncoding RNAs with a length of 19–24 nucleotides [4]. Through binding to the 3′-untranslated regions (3′-UTRs) of their targets, miRs can promote RNA degradation or repress mRNA translation involved in tumor cell proliferation, invasion and metastasis [5–7]. Accumulating literatures have indicated that miRs have been widely proposed as tumor suppressors or oncogenes in different cancers. For instance, miR-188-5p has been reported to positively contribute to the tumorigenesis and development of gastric cancer by down-regulating PTEN and activating Wnt/β-catenin signaling pathway [8]. Jiang et al. revealed a tumor suppressive role of miR-449b-5p that restricts the growth and invasion of breast cancer cells through inhibiting CREPT-mediated activation of Wnt/β-catenin [9]. In addition, miR-582-5p exerts oncogenic role in prostate cancer [10] and tumor suppressive role in colorectal cancer [11], which indicated its dual function against different tumors. Recently, miR-924 was shown to inversely regulate the expression of α-defensin 5 (DEFA5) in inflammatory bowel disease [12]. Interestingly, lncRNA n335586 promoted hepatocellular carcinoma cell migration and invasion through facilitating the expression of its host gene CKMT1A by competitively binding miR-924 in HBV-related hepatocellular carcinoma [13]. Nevertheless, it is still unclear whether miR-924 plays a role as a tumor suppressor gene or an oncogene in NSCLC.

Rhomboid domain-containing protein 1 (RHBDD1), a new member of the Rhomboid family is highly responsible for the regulation of apoptosis cleaving pro-apoptotic Bcl-2 family protein BIK [14]. Related studies showed that RNA interference (RNA) mediated RHBDD1 silencing significantly suppressed cell proliferation and cell cycle progression in hepatocellular carcinoma [15], glioblastoma [16] and colorectal cancer [17]. Moreover, Zhang et al. [18] demonstrated that RHBDD1 could promote tumor metastasis by promoting Wnt/β-catenin signaling and epithelial–mesenchymal transition in colorectal cancer. Similar results were also observed in breast cancer by Zhang et al. [19] and Huang et al. [20]. In view of this, we wonder the effects and molecular mechanisms of RHBDD1 on NSCLC, which have not been reported until now.

In this study, we analyzed the expression of miR-924 and its clinicopathological implication in NSCLC as well as the expression of RHBDD1 and its prognostic value in lung cancer patients. Using gain-of-function and loss-of-function assays, we analyzed the function role of miR-924 and RHBDD1 on NSCLC cells in vitro and in vivo. Furthermore, we further validate whether miR-924 regulated the tumorigenesis of NSCLC by modulating the RHBDD1/Wnt/β-catenin signaling pathway.

## Materials and methods

### Clinical tissue collection

A total of 79 cases of NSCLC tissue samples and paired adjacent tissues were collected at The First Hospital Of China Medical University (Shenyang, China) between January 2014 and December 2017. All the participants signed the written informed consent and were confirmed not to receive any radiotherapy or chemotherapy before the lung resection operation. Specimens were immediately frozen in liquid nitrogen and stored at − 80 °C freezer until further analysis. The basic clinicopathological characteristics of NSCLC patients were summarized in Table 1. The present study was performed in accordance with the guidelines of the Declaration of Helsinki and obtained the approval from Ethics Committee of The First Hospital Of China Medical University (Shenyang, China).

**Table 1:** Correlation of miR-924 with clinicopathological features of patients with non-small cell lung cancer

miR-924 expression				
Variable	Total (n = 79)	High (n = 34)	Low (n = 45)	P value
Gender				1.012
Male	62	26	36	
Female	17	8	9	
Age (year)				0.589
< 60	45	13	32	
≥ 60	34	21	13	
Smoking history				0.485
Yes	51	24	27	
No	28	10	18	
Tumor size (cm)				0.063
< 4	28	17	11	
≥ 4	51	17	34	
TNM stage				*0.003*
I + II	66	30	36	
III + IV	13	4	9	
Lymphatic metastasis				*0.016*
Yes	28	7	21	
No	51	27	24	
Pathological type				0.886
Adenocarcinoma	36	15	21	
Squamous carcinoma	43	19	24	

The italic values indicates the *P* values which are less than 0.05

### Cell culture

Total four NSCLC cell lines (95D, H1299, A549 and H520) and normal human bronchial epithelial cell lines (BEAS-2B) were obtained from American Type Culture Collection (ATCC, Manassas, VA, USA). A549 and H520 cells were cultured in RIMI-1640 medium (HyClone, GE Healthcare Life Sciences, MA, USA). 95D, H1299 and BEAS-2B cells were cultured in DMEM (HyClone). All culture media were supplemented with 10% fetal bovine serum (FBS, Gibco) and maintained in a humidified atmosphere containing 5% CO_2_ at 37 °C.

### Quantitative real time PCR

Total RNA was isolated from tissues or cultured cells using TRIzol reagent (Takara Bio Inc., Shiga, Japan). The reverse transcription was carried out using ABI’s TaqMan MicroRNA Reverse Transcription kit (Applied Biosystems, Thermo Fisher Scientific). Quantitative real-time PCR was performed to detect miR-924 expression level on Applied Biosystems 7900HT Sequence Detection system using a Taqman MicroRNA assay (Applied Biosystems) under the following reaction conditions: 95 °C for 10 min, 92 °C for 15 s, and 60 °C for 1 min, for 40 cycles. Relative miR-924 expression was derived using the 2^−ΔΔCT^ method with U6 as an internal control.

### Cell transfection

Human synthetic miR-924 mimic and negative control miRNA mimic (miR-NC) were purchased from RiboBio Co., Ltd., (Guangzhou, China). Small interfering RNA targeting RHBDD1 (si-RHBDD1) and a corresponding NC (si-NC) were purchased from GenePharma Co. Ltd., (Shanghai, China). RHBDD1 overexpression plasmid was generated by inserting the full-length human RHBDD1 cDNA into the mammalian expression vector pcDNA3.1 (Invitrogen, Carlsbad, CA, USA) by GenePharma Co. Ltd., (Shanghai, China). Meanwhile, the empty vector was used as a control. H1299 or A549 cells were plated in 6-well plates at the concentration of 3.0 × 10^6^ and transfected with the above nucleotides according to the instructions of Lipofectamine 2000 (Invitrogen) for 48 h.

### Cell proliferation assay

Transfected cells were seeded into 96-well plates at a density of 3.0 × 10^3^/well and cultured indicated time points (24, 48 and 72 h, respectively) at 37 °C. Then, 10 µl Cell Counting Kit-8 (CCK-8, Sigma-Aldrich) was added to each well at the above time points and cells were incubated for another 2 h at 37 °C. Subsequently, the absorbance at the wavelength of 450 nm was read using a microplate reader (Bio-Tek Company, Winooski, VT, USA).

### Transwell assays

Transwell assays were performed using a 24-well chamber (8-µm-pore size, Corning, NY, USA) without (for cell migration) or with Matrigel (for invasion). In brief, 300 µl of a cell suspension in FBS-free medium containing 5 × 10^4^ transfected cells were seeded in the upper chamber. At the same time, 700 µl of growth medium containing 10% FBS as was placed in the lower chamber to stimulate cell migration or invasion. After 24 h incubation in a 37 °C incubator, cells that migrated to the lower chamber were fixed with 4% paraformaldehyde and stained with 0.5% crystal violet. Subsequently, the migrated and invaded cells were photographed and counted in five randomly selected five fields under an inverted light microscope (magnification, 200×).

### Luciferase reporter assay

The gene sequence of RHBDD1 binding to miR-924 was predicted by miRNA target prediction program (TargetScan: http://www.targetscan.org/vert_71/). The wild-type (WT) and mutant (MUT) 3′UTR of RHBDD1, containing putative and mutated miR-924 binding sites, respectively, were chemically synthesized by RiboBio Co., Ltd., (Guangzhou, China) and inserted into the psiCHECK-2 vector (Promega, Madison, USA). For luciferase reporter assay, H1299 or A549 cells were seeded into 24-well plates and co-transfected with miR-924 mimic or miR-NC and WT RHBDD1 or MUT RHBDD1 using Lipofectamine 2000 reagent (Invitrogen). At 48 h post-transfection, cells were collected and luciferase activities were determined using Dual-Luciferase Reporter Assay System (Promega, Madison, USA).

### Oncomine meta-analysis

A meta-analysis based on Oncomine database (www.onocomine.org) was performed to compare the expression profile of RHBDD1 between lung cancer tissues and normal lung tissues. The following key terms were set in this meta-analysis: “RHBDD1”, “Cancer vs. Normal Analysis”, “Lung Cancer” and “mRNA”.

### Kaplan–Meier plot analysis

The prognostic value of RHBDD1 gene in lung cancer was analyzed using Kaplan–Meier Plotter (http://kmplot.com/analysis/), a database that integrates gene expression data and clinical data [21]. Here, we focused our analysis on overall survival patient information by dividing all patient samples into two groups (higher and lower RHBDD1 expression) based on the medium value. Then, we calculated the hazard ratio with 95% confidence the hazard ratio with 95% confidence intervals and log rank p value with p value less than 0.01 as a threshold.

### Tumor xenograft experiment

Six-week-old male BALB/C nude mice were obtained from Shanghai Laboratory Animals Center of Chinese Academy of Sciences (Shanghai, China) and maintained in pathogen-free environment with controlled temperature. For tumorigenic studies, H1299 cells with stable overexpression of miR-924 or miR-NC (2 × 10^6^ cell per mouse) were subcutaneously inoculated into the flanks of nude mice. Accordingly, these mice were classified into two groups, including miR-924 mimic and miR-NC group (n = 04 mice per group). Tumor volumes in each group were measured by digital caliper at 5 days internal from day 5 using the formula: V = (L × W^2^)/2, where L was the length and W was the width of the tumor. After 30 day observation, the mice were sacrificed by cervical dislocation and the xenograft tumors were resected and weighed. Afterwards, tumor samples were collected for quantitative real time PCR and western blot analysis. All animal experiments were approved by the Animal Ethics Committee and undertaken in accordance with the Care and Use of Laboratory Animals of The First Hospital Of China Medical University (Shenyang, China).

### Western blot analysis

Protein samples were prepared with RIPA lysis buffer (Beyotime, Shanghai, China) and separated by 12% sodium dodecyl sulfate-polyacrylamide gel electrophoresis. Afterwards, the protein bands in the gel were transferred onto polyvinylidene difluoride membranes (Millipore, MA, USA). Membranes were blocked with 5% fat-free milk and incubated with primary antibodies against RHBDD1, Wnt1, β-catenin, GSK-3β, p-GSK-3β, E-cadherin, Vimentin and GAPDH (All from Abcam, Cambridge, UK) at 4 °C overnight, followed by incubation with horseradish peroxidase-conjugated secondary antibodies at room temperature for 2 h. The protein signals were visualized by enhanced chemiluminescence solution (Pierce; Thermo Fisher Scientific, Inc.).

### Statistical analysis

Statistical analysis was performed using SPSS 19.0 (SPSS Inc., Chicago, IL, USA). All the experiments were repeated three times independently. Data were expressed as mean ± standard deviation. The Chi-squared test was used to evaluate the correlation between miR-924 levels and clinicopathological parameters in patients with NSCLC. Differences were analyzed with Student’s t-test between two groups and one-way analysis of variance, followed by Dunnett’s test for multiple groups. A statistically significant difference was accepted when *p*-values less than 0.05.

## Results

### MiR-924 was significantly down-regulated in NSCLC tissues and cell lines

**Fig. 1:**
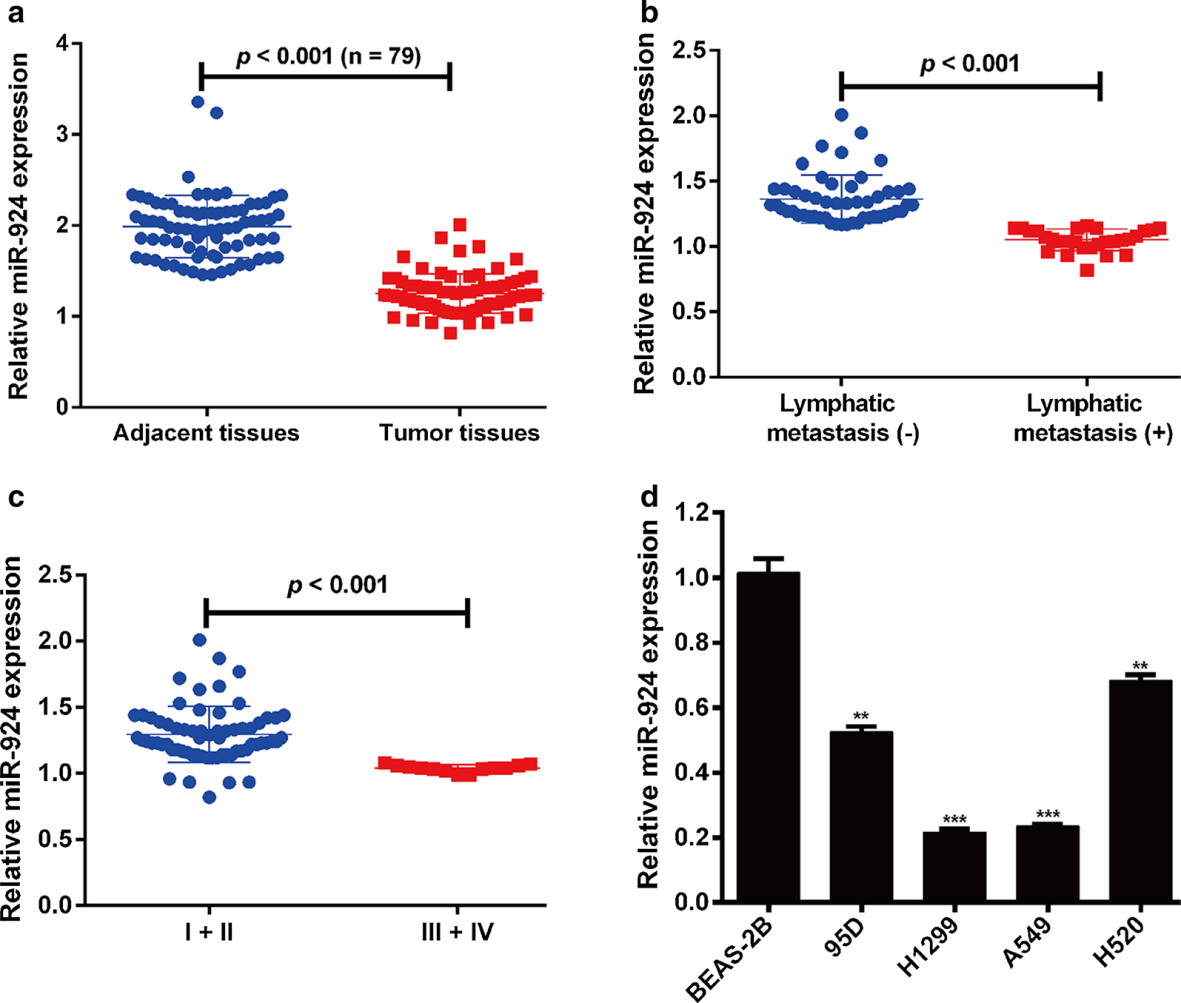
Decreased miR-924 expression in NSCLC tissues and cell lines. **a** Quantitative real time PCR was used to determine the expression profile of miR-924 in NSCLC tissues and adjacent tissues from 79 patients. NSCLC patients with metastasis (**b**) or advanced TNM stage (**c**) had lower miR-924 expression levels compared with those of non-metastatic or early TNM stage. The miR-665 expression level was assessed in four NSCLC cell lines (95D, H1299, A549 and H520) and normal human bronchial epithelial cell line BEAS-2B. **p < 0.01, ***p < 0.001, compared with BEAS-2B

The expression level of miR-924 was measured in 79 pairs of frozen NSCLC tissues and adjacent lung tissues using quantitative real time PCR. As shown in Fig. 1a, the expression level of miR-924 was significantly lower in NSCLC tissues than in adjacent normal tissues. Patients were then divided into high miR-924 expression (n = 34) and low miR-924 expression (n = 45) groups using the median value of miR-924 in NSCLC tissues as the cutoff. The expression of miR-924 in NSCLC tissues was significantly inversely correlated with lymphatic metastasis (*p* = 0.016) and clinical TNM stage (*p* = 0.003). In other words, patients who had a lower miR-924 expression in tumor tissues had a higher probability of lymphatic metastasis and a more advanced TNM classification (Table 1). Additionally, patients who had lymphatic metastasis (Fig. 1b) or advanced TNM classification (Fig. 1c) had a lower miR-924 expression level. Furthermore, we determined miR-924 expression in NSCLC cell lines (95D, H1299, A549 and H520) and normal human bronchial epithelial cell line BEAS-2B. Consistently, lower miR-924 expression was observed in all four NSCLC cell lines when compared with that in BSAS-2B cells (Fig. 1d). These results indicate that down-regulation of miR-924 might play a critical role in the malignant development of NSCLC.

### MiR-924 inhibited cell proliferation, migration and invasion of NSCLC

**Fig. 2:**
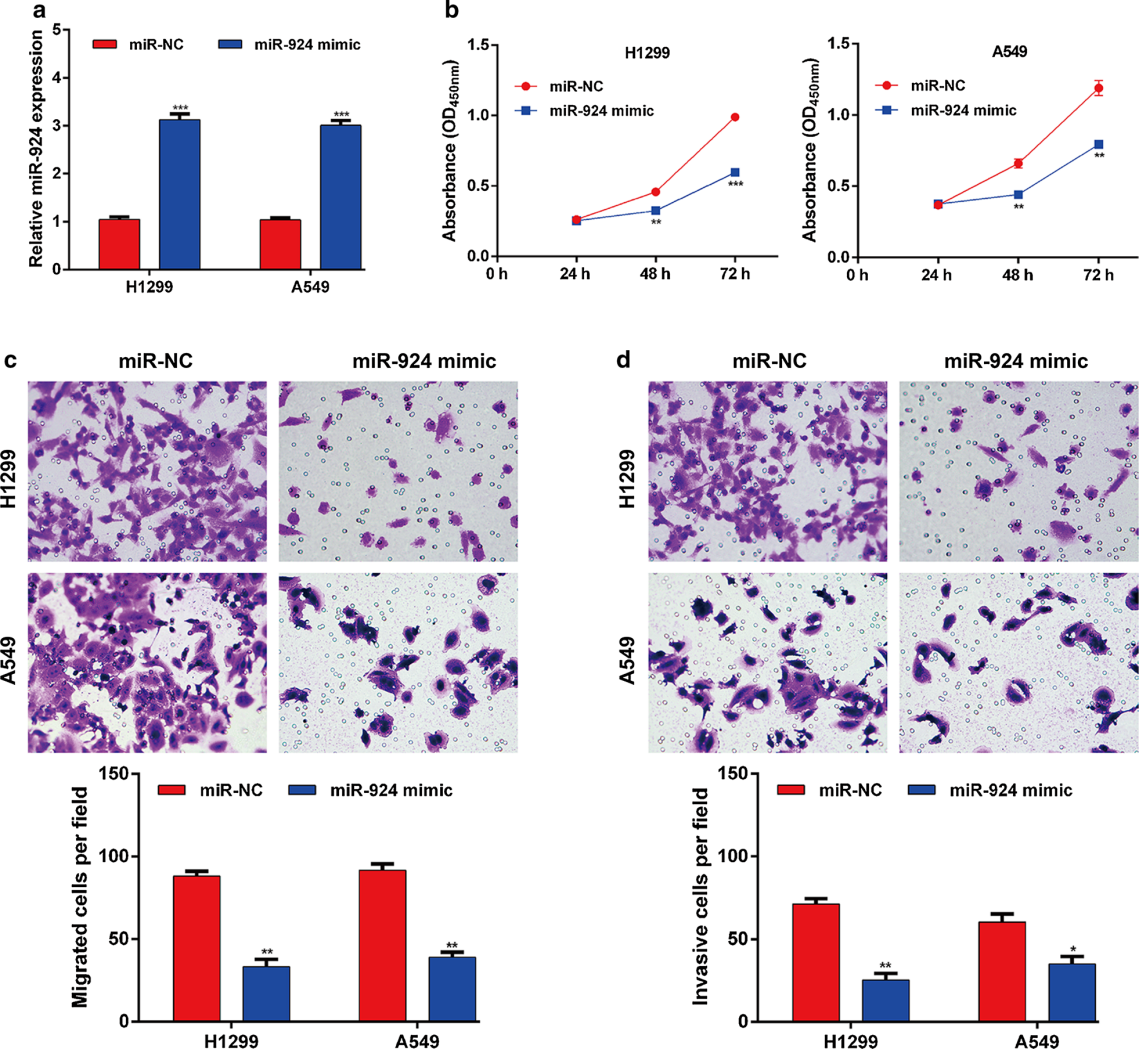
MiR-924 suppressed the proliferation, migration and invasion of NSCLC cells. **a** MiR-924 expression levels in miR-924 mimic or miR-NC transfected H1299 and A549 cells. **b** Cell proliferation was assessed by CCK-8 assay in miR-924 mimic or miR-NC transfected H1299 and A549 cells. **c** Representative images and quantification of the Transwell migration assay in miR-924 mimic or miR-NC transfected H1299 and A549 cells. **d** Representative images and quantification of the Transwell invasion assay in miR-924 mimic or miR-NC transfected H1299 and A549 cells. *p < 0.05, **p < 0.01, ***p < 0.001, compared with miR-NC

We next explore the functional role of miR-924 in NSCLC in vitro by performing gain-of-function assays in H1299 and A549 cells, which exhibited the lowest miR-924 expression among the four tested NSCLC cell lines. The constructed miR-924 mimic or miR-NC was successfully transferred into H1299 and A549 cells to cause overexpression of miR-924 expression, which was confirmed by quantitative real time PCR (Fig. 2a). Then CCK-8 and transwell assays were carried out to assess the role of miR-924 in NSCLC cell proliferation, migration and invasion. As depicted in Fig. 2b, transfection of H1299 and A549 cells with the miR-924 mimic significantly decreased cell proliferation ability, compared with miR-NC transfection. In addition, H1299 and A549 cells with elevated expression levels of miR-924 had fewer migratory cells in the transwell migration assay (Fig. 2c). Similarly, miR-924 overexpression significantly suppressed the cell invasive ability in both H1299 and A549 cells (Fig. 2d). Together, these results indicated that miR-924 impairs the proliferation and metastatic capacity of NSCLC cells.

### RHBDD1 was a direct target of miR-924 in NSCLC cells

**Fig. 3:**
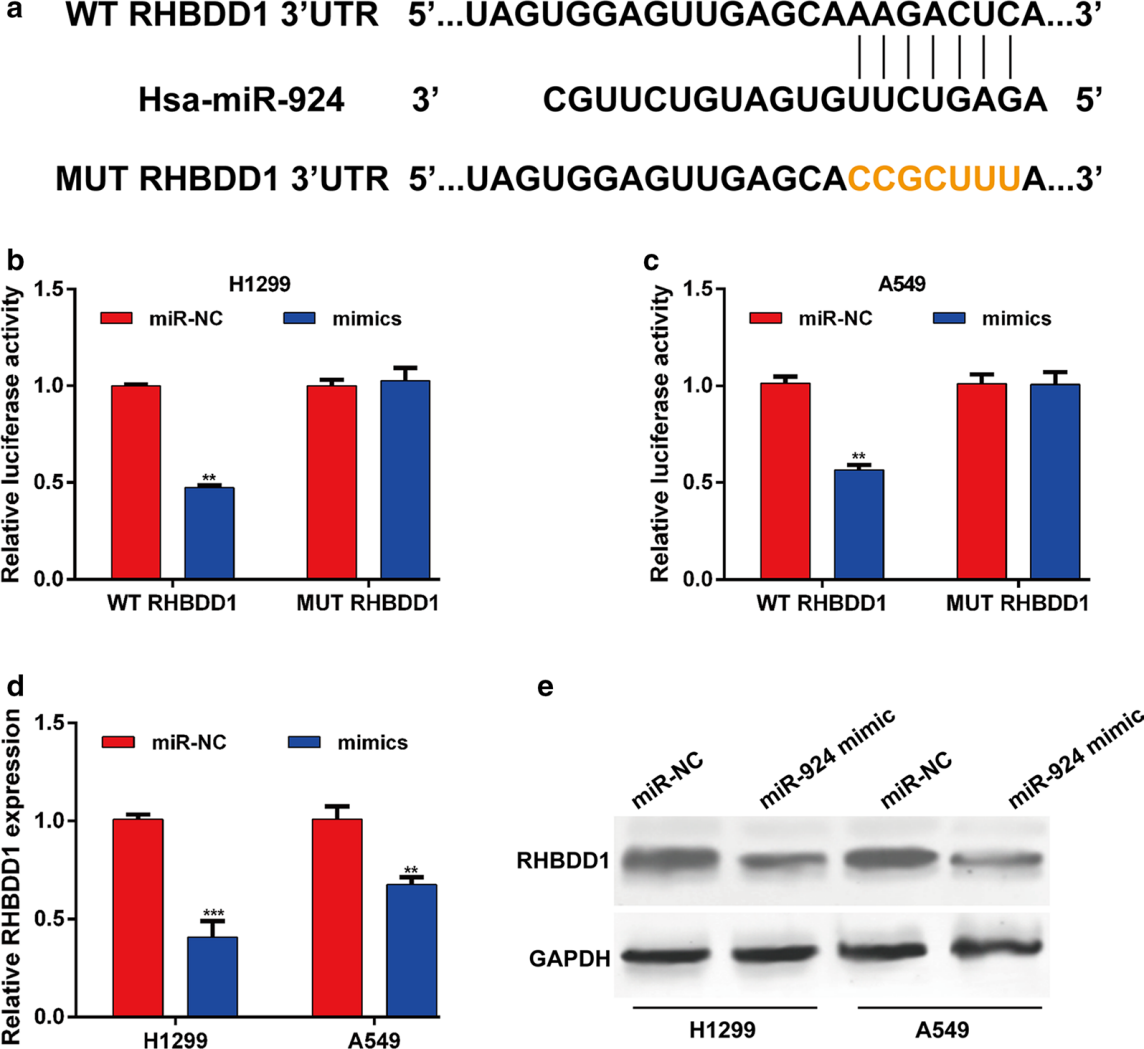
Identification of RHBDD1 as a direct target gene of miR-924 in NSCLC cells. **a** Bioinformatics prediction revealed a highly conserved miR-924 binding site in the 3′-UTR of RHBDD1. **b**, **c** H1299 or A549 cells were transfected with either miR-924 mimic or miR-NC and either wild-type (WT) or mutant (MUT) 3′-UTR RHBDD1 reporter plasmid. Luciferase activity was measured at 48 h post-transfection and normalized to that of the Renilla luciferase activity. **d**, **e** Expression levels of RHBDD1 mRNA and protein in miR-924-overexpressing H1299 and A549 cells were measured by using quantitative real time PCR and western blot analysis, respectively. **p < 0.01, ***p < 0.001, compared with miR-NC

Subsequently, we predicted the putative targets of miR-924 were predicted via open access database TargetScan (http://www.targetscan.org/). Among the predicted target genes, RHBDD1 was of special interest to us for it was reported to be tumor cell metastasis. As shown in Fig. 3a, the 3′UTR of RHBDD1 contained a highly conserved binding site for miR-924. Subsequently, a luciferase reporter assay was conducted to verify this prediction in NSCLC cells. The results showed the relative luciferase activity of RHBDD1 3′UTR was markedly suppressed upon the overexpression of miR-924 in H1299 (Fig. 3b) and A549 (Fig. 3c) cells. In contrast, the luciferase activity of a plasmid containing a mutant RHBDD1 3′-UTR was unaffected by miR-924 overexpression. Moreover, quantitative real time PCR (Fig. 3d) and western blot analysis (Fig. 3e) further demonstrated that miR-924 overexpression significantly down-regulated the expression of RHBDD1 mRNA and protein levels in both H1299 and A549 cells. Collectively, above data demonstrated that RHBDD1 might be a direct downstream target of miR-924 in NSCLC cells.

### RHBDD1 was up-regulated in NSCLC tissues and inversely associated with prognosis

**Fig. 4:**
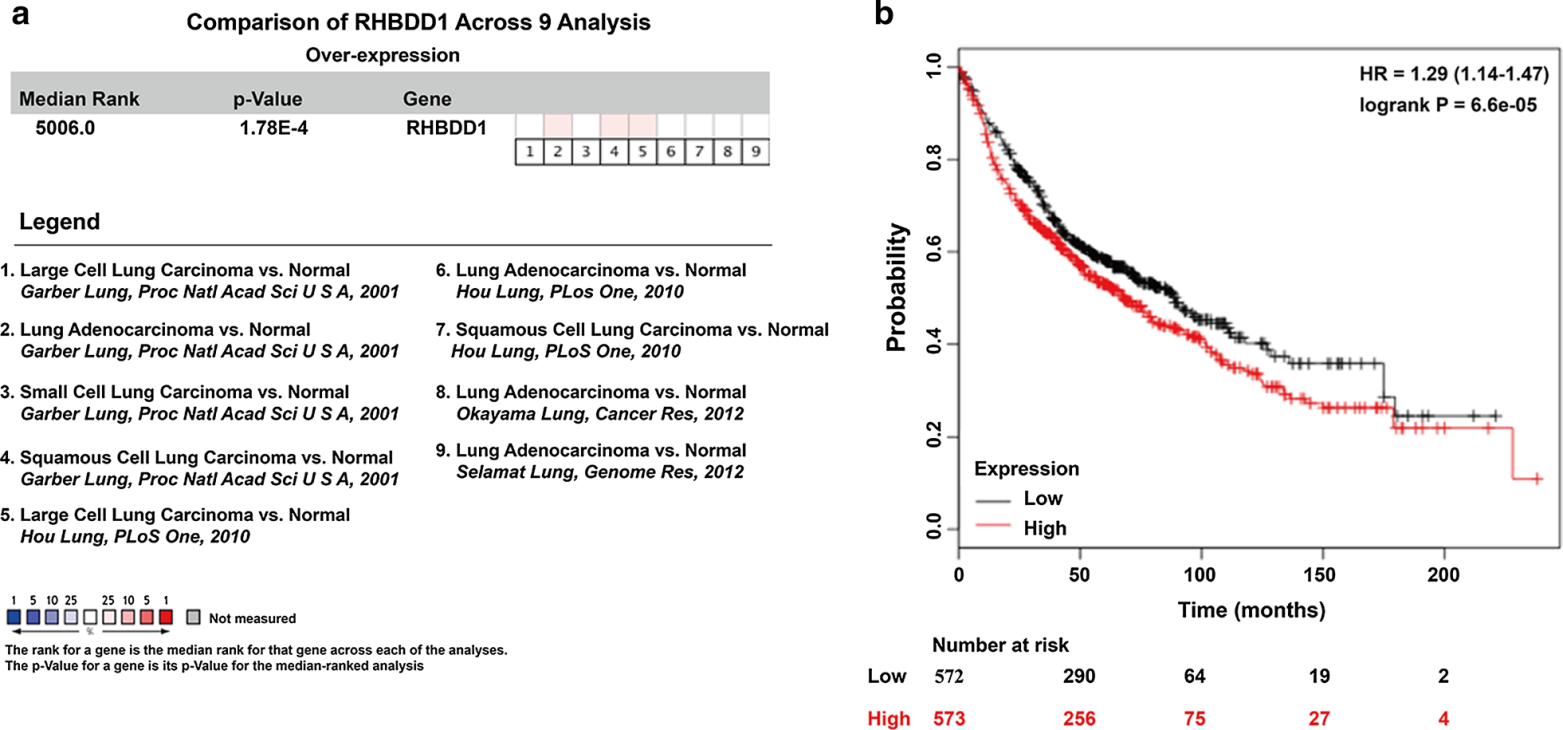
Up-regulated RHBDD1 was inversely associated with prognosis in NSCLC. **a** Total four Oncomine microarray datasets regarding RHBDD1 mRNA expression in lung cancer vs. normal tissues were included in our meta-analysis. Data are shown as the median rank of RHBDD1 through each dataset analysis. p-value for RHBDD1 was presented using the median ranked analysis on lung cancer vs. normal tissues. **b** Kaplan–Meier plots showing the effects of RHBDD1 on 5-year overall survival in NSCLC. In red: patients with expression above the median and in black, patients with expressions below the median

To investigate the expression pattern of RHBDD1 in lung cancer, meta-analysis of RHBDD1 gene expression was performed using public microarray datasets form Oncomine database. Total four online microarray datasets were included our analysis (Fig. 4a), which consistently indicated that the expression of RHBDD1 mRNA was significantly up-regulated in lung cancer tissues compared with the normal lung tissues (gene median rank: 5006.0, *p* = 1.78E−4). Moreover, the prognostic value of RHBDD1 in lung cancer patients was analyzed using a public database Kaplan–Meier Plotter (http://kmplot.com/analysis/). As shown in Fig. 4b, NSCLC patients with higher RHBDD1 expression (median survival time: 76.5 month) in NSCLC tissues had much shorter overall survival rates compared with those of NSCLC patients with lower RHBDD1 expression (median survival time: 94.3 month). These analyses suggested that RHBDD1 might promote the development and progression of NSCLC.

### Biological functions of miR-924 driven in NSCLC cells by RHBDD1 inhibition

**Fig. 5:**
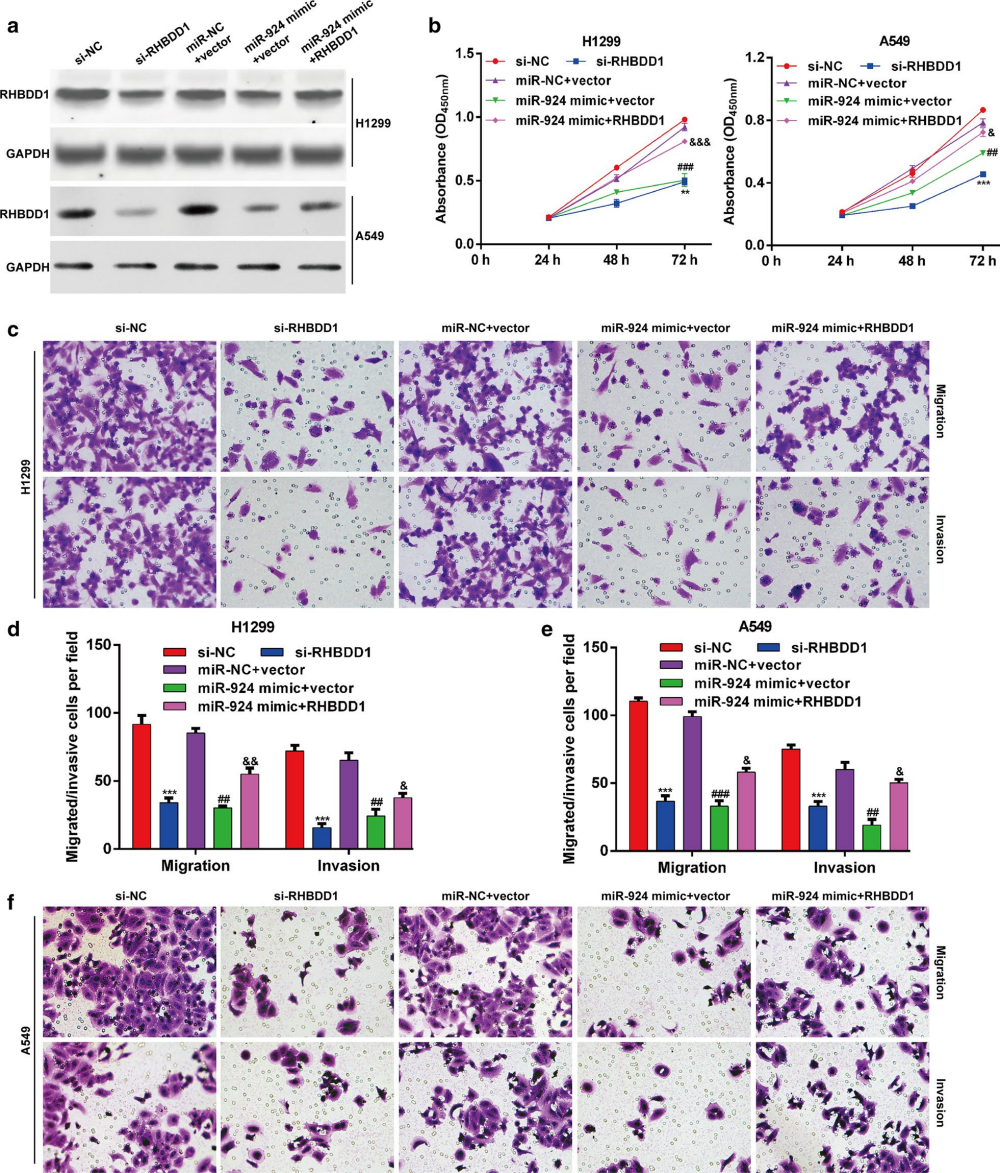
MiR-924 inhibited NSCLC cell proliferation, migration and invasion by suppressing RHBDD1. H1299 or A549 cells were transfected with si-NC, si-RHBDD1, miR-924 mimic or miR-924 mimic plus RHBDD1, respectively. **a** The protein level of RHBDD1 expression was detected by western blot analysis. **b** Cell proliferation was analyzed in transfected H1299 or A549 cells as above. Transwell assay was performed to evaluate cell migration and invasion abilities in transfected H1299 (**c**, **d**) or A549 (**e**, **f**) cells. **p < 0.01, ***p < 0.001, compared with si-NC; ^##^*p* < 0.01, ^###^*p* < 0.001, compared with miR-NC + vector; ^&^*p* < 0.05, ^&&^*p* < 0.01, ^&&&^*p* < 0.001, compared with miR-924 mimic + vector

To investigate whether miR-924 inhibited NSCLC cell proliferation, migration and invasion by mediating RHBDD1, H1299 or A549 cells were selected to transfect with si-NC, si-RHBDD1, miR-924 mimic or miR-924 mimic plus RHBDD1, respectively. Western blot analysis showed that RHBDD1 protein expression was obviously decreased after si-RHBDD1 transfection and reduced RHBDD1 protein by miR-924 mimic transfection was partially recovered by RHBDD1 overexpression in both H1299 and A549 cells (Fig. 5a). Then, CCK-8 assay (Fig. 5b) showed that RHBDD1 knockdown significantly suppressed the proliferation ability in H1299 and A549 cells. It was noteworthy that restoration of RHBDD1 could partially recover the inhibitory effects of miR-924 overexpression on cell proliferation in H1299 and A549 cells (Fig. 5b). In addition, RHBDD1 knockdown imitated, while overexpression reversed the suppressive role of miR-924 overexpression on cell migration and invasion in H1299 (Fig. 5c, d) and A549 (Fig. 5e, f) cells. Overall, our data demonstrated that miR-924 impaired the proliferation and migratory ability of NSCLC cells, at least in part, through repressing the expression of RHBDD1.

### MiR-924 suppressed the Wnt/β-catenin signaling pathway in NSCLC cells via repressing RHBDD1

**Fig. 6:**
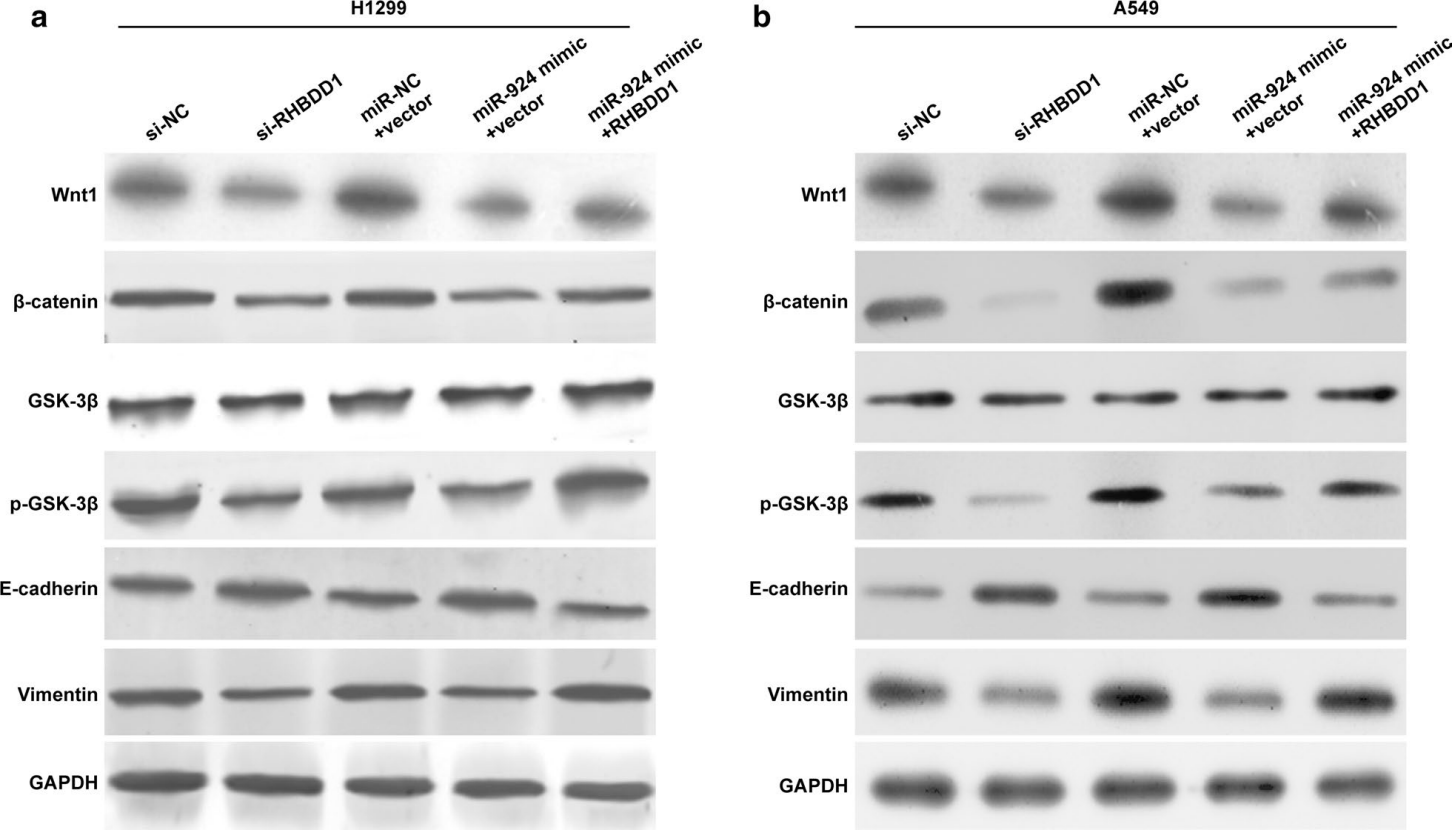
MiR-924 negatively regulated the Wnt/β-catenin pathway by directly targeting RHBDD1. H1299 or A549 cells were transfected with si-NC, si-RHBDD1, miR-924 mimic or miR-924 mimic plus RHBDD1, respectively. Western blot assay was used to compare the expression level of Wnt1, β-catenin, GSK-3β, p-GSK-3β, E-cadherin and vimentin in transfected H1299 (**a**) and A549 (**b**) cells. GAPDH was used as an internal control

The Wnt/β-catenin signaling pathway has previously been reported to be tumor cell proliferation, migration and invasion. We further investigated whether Wnt/β-catenin signaling pathway involved in the regulation of NSCLC cells induced by miR-924 targeting RHBDD1. As shown in Fig. 6a, RHBDD1 knockdown or miR-924 overexpression obviously suppressed the expression of Wnt1, β-catenin and phosphorylated GSK-3β in H1299 cells. In addition, we detected the expression of EMT-marker protein and found that RHBDD1 knockdown or miR-924 overexpression markedly up-regulated E-cadherin expression, but down-regulated vimentin expression. Notably, recovery of RHBDD1 expression in miR-924 overexpressed H1299 cells partially alleviated the changes in the expression of Wnt1, β-catenin, p-GSK-3β, E-cadherin and vimentin caused by miR-924 overexpression solely. The similar expression levels of Wnt1, β-catenin, phosphorylated GSK-3β, E-cadherin and vimentin were also observed in A549 cells (Fig. 6b). These results indicated that RHBDD1 was a direct target gene of miR-924 involved in Wnt/β-catenin pathway in NSCLC cells.

### MiR-924 inhibited tumor growth in vivo by inhibiting RHBDD1/Wnt/β-catenin signaling pathway

**Fig. 7:**
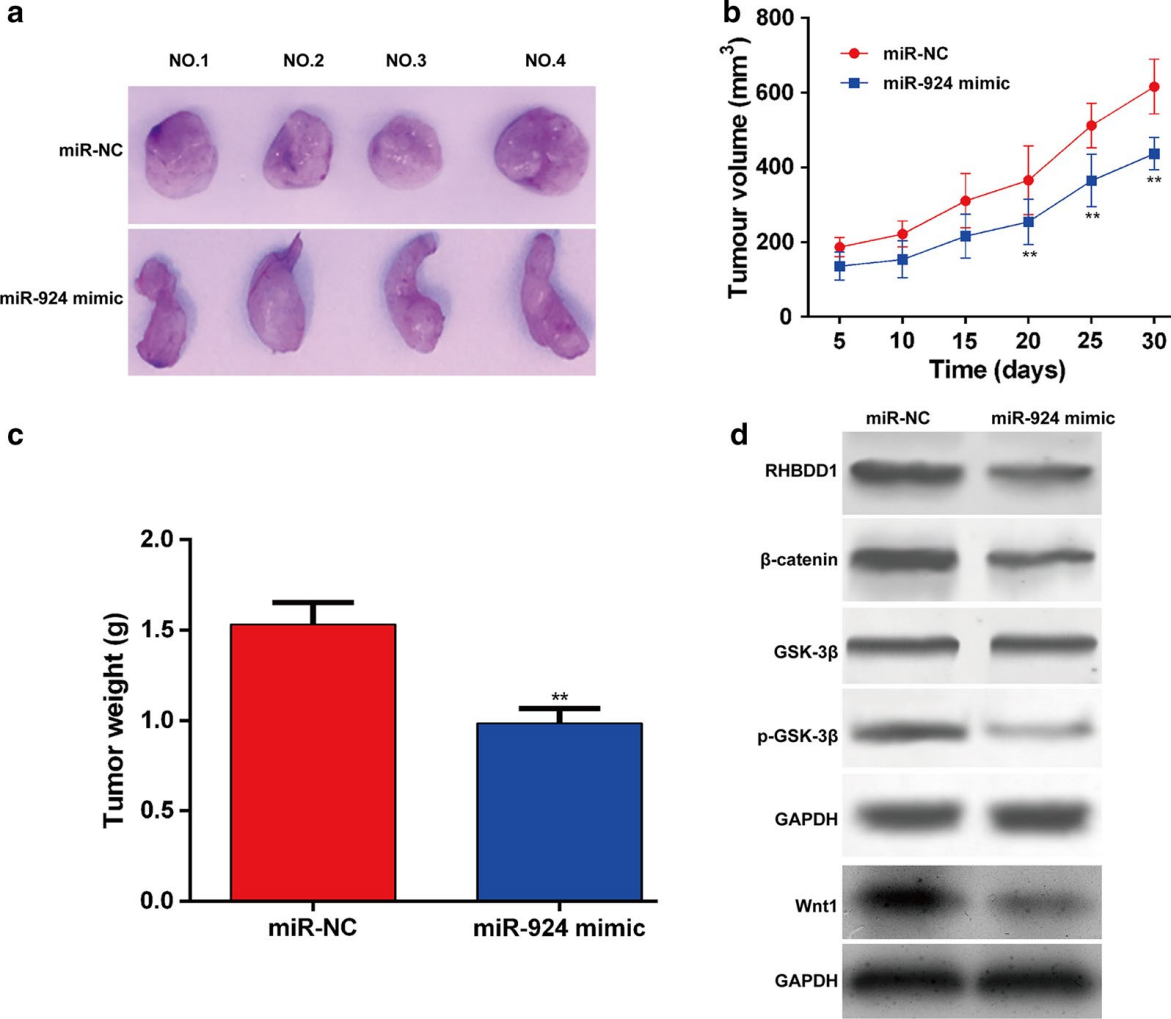
MiR-924 inhibited tumor growth of NSCLC in vivo by targeting RHBDD1 via deactivation of the Wnt/β-catenin pathway. **a** Representative images of xenograft tumors excised from nude mice derived 30 days after subcutaneous inoculation of H1299 cells transfected with miR-924 mimic or miR-NC. **b** The volume of tumor xenografts was measured every 5 days. The growth of the tumor xenografts in the miR-924 mimic group was notably slower than that in the miR-NC group. **c** All nude mice were sacrificed 30 days after injection, and the tumor xenografts were excised. The weight of the tumor xenografts in the miR-924 mimic group was notably lower than that in the miR-NC group. **d** The protein levels of RHBDD1, Wnt1, β-catenin, GSK-3β and p-GSK-3β in xenografts were examined using western blot. **p < 0.01, compared with miR-NC

To further illustrate whether miR-924 inhibited the tumorigenesis of NSCLC in vivo, we established a tumor xenograft mouse model by subcutaneously inoculating H1299 cells transfected with miR-924 mimic or miR-NC into the flanks of nude mice. As shown in Fig. 7a, the tumor xenografts generated from the miR-924 mimic-transfected cells were obviously smaller than the xenografts in mice of the miR-NC group. Moreover, the time-dependent analysis showed that the tumor volume was increased more slowly in miR-924 mimic group than that in miR-NC group (Fig. 7b). The tumor xenografts generated from the miR-924 mimic-transfected cells were significantly lighter than that from miR-NC-transfected cells (Fig. 7c). Furthermore, we detected the expression of RHBDD1, Wnt1, β-catenin and p-GSK-3β in xenografts through western blot. Consistent with the in vitro experiments, the expression levels of RHBDD1, Wnt1, β-catenin and p-GSK-3β were down-regulated in the tumor xenografts of mice in the miR-924 mimic group compared with miR-NC group (Fig. 7d). Above results suggested that miR-924 might serve as a tumor suppressor by targeting RHBDD1 via deactivation of the Wnt/β-catenin pathway in NSCLC.

## Discussion

Increasing evidence indicate the important role of miRNAs as key regulators in tumorigenesis and progression by modulating tumor cell proliferation and metastasis via targeting tumor suppressive genes or oncogenes [22]. In this study, we collected 79 matched NSCLC tissue and normal lung tissue samples and demonstrated that miR-924 was expressed at a lower level in the NSCLC tissues. Moreover, the relative lower expression level of miR-924 in NSCLC tissues was significantly associated with lymphatic metastasis, clinical TNM stage and overall survival. These results are consistent with those from previous studies that show miR-924 was decreased in inflammatory bowel disease [12] and hepatocellular carcinoma [13].

In vitro and in vivo experiments verified that miR-924 suppressed cell proliferation, migration, invasion and tumor growth. Our team further demonstrated that miR-924 inhibited the activity of β-catenin and p-GSK-3β, as key effector of Wnt/β-catenin signaling pathway and inactivated EMT markers (E-cadherin and vimentin). It has been widely accepted that Wnt/β-catenin signaling pathway exerts fundamental mechanisms in cell proliferation and invasion, which was activated in multiple types of tumors [23, 24]. In addition, the Wnt/β-catenin pathway is closely relates to EMT and plays a critical role in metastasis [25]. Similar with our data, Zhang et al. showed that miR-770 overexpression was capable of inhibiting NSCLC tumor growth by inhibiting its downstream WNT/β-catenin pathway both in vitro and in vivo [26]. Down-regulation of miR-3127-5p promotes EMT through activating the Wnt/FZD4/β-catenin signaling pathway in NSCLC [27].

Generally, miRNAs exert their functions by binding to the 3′UTR of their downstream target genes [28, 29]. Recently, it has been reported that miR-924 has two targets in different diseases, including CKMT1A in hepatocellular carcinoma [13] and α-defensin 5 (DEFA5) in inflammatory bowel disease [12]. Here, RHBDD1 was identified and confirmed as a direct target of miR-924 in NSCLC cells. Using public microarray datasets, higher RHBDD1 expression in NSCLC patients had much shorter overall survival rates. Although RHBDD1 as a target gene by miRNAs was rarely reported in diseases, RHBDD1 has been frequently confirmed to be highly expressed in human cancers, such as colorectal cancer, glioblastoma, hepatocellular carcinoma, and chronic myeloid leukemia, whose knockdown inhibits the proliferation of these tumor cells [15, 16, 30, 31]. Our data further demonstrated that RHBDD1 knockdown imitated, while overexpression reversed the effects of miR-924 on NSCLC cell proliferation, migration and invasion, as well as Wnt/β-catenin signaling pathway. Our findings are in accordance with previous report, suggesting that RHBDD1 can activate Wnt/β-catenin signaling pathway to promote tumor growth and metastasis in colorectal cancer [18]. Additionally, some limitations, including smaller sample size, lacking of loss-of-function assay, more NSCLC cell functional assays and deeper investigation on molecular mechanisms were existed in our study, which will be our next work goal.

## Conclusions

In summary, our present study suggested that miR-924 function as a tumor suppressor in NSCLC cells by inhibiting cell proliferation, migration and invasion, at least in part, be ascribed to the negative regulation of RHBDD1 through Wnt/β-catenin signaling pathway. These results suggest that miR-924 might be a novel therapy target for diagnosis and prognosis of NSCLC.

## Data Availability

All data generated or analyzed during this study are included in this published article.

## Abbreviations

NSCLC: Non-small cell lung cancer
TNM: Tumor-node-metastasis
RHBDD1: Rhomboid domain-containing protein 1
3′-UTR: 3′-untranslated region
ATCC: American Type Culture Collection
FBS: Fetal bovine serum
NC: Negative control
CCK-8: Cell Counting Kit-8
